# Acute Esophageal Necrosis (Black Esophagus) in the Setting of Cardiac Arrhythmia: A Case Report

**DOI:** 10.7759/cureus.80970

**Published:** 2025-03-21

**Authors:** Soukaina Essadiqi, Omar Bahlaoui, Anass Nadi, Wafaa Khannoussi, Imane Ben El Barhdadi

**Affiliations:** 1 Mohammed VI Faculty of Medicine, Mohammed VI University of Sciences and Health, Casablanca, MAR; 2 Gastroenterology Department, Cheikh Khalifa International University Hospital, Casablanca, MAR; 3 Gastroenterology Department, Mohammed VI International University Hospital, Casablanca, MAR

**Keywords:** atrial fibrillation, esophageal necrosis, hematemesis, ppi, upper gi endoscopy, visceral ischemia

## Abstract

Acute esophageal necrosis is a rare (0.01%) and life-threatening condition (5% specific-mortality) involving blackening of the esophagus mucosa resulting from a combination of ischemic damage and gastric acid reflux, although the exact pathophysiology is still unclear. A 70-year-old female patient was admitted to the intensive care unit following two episodes of hematemesis. Her medical history included diabetes mellitus and ischemic heart disease. At admission an inaugural complete atrial fibrillation was discovered on electrocardiogram. Biological and bacteriological tests revealed urinary tract infection. An esophageo-gastro-duodenoscopy showed typical blackening of the lower third of the esophagus. Treatment consisted of intravenous fluids, high-dose proton pump inhibitors, nothing through the mouth status and intravenous antibiotic therapy. Repeat esophageo-gastro-duodenoscopy showed significant improvement. However, her condition deteriorated as she succumbed to septic shock. Our patient presented with negative prognostic factors like advanced age, diabetes mellitus, ischemic heart disease and inaugural arrhythmia that may have contributed to the development of acute esophageal necrosis.

## Introduction

Acute esophageal necrosis (AEN) is a rare and life-threatening condition, characterized by a striking, circumferential blackening of the esophageal mucosa, occurring in approximately 0.01% of cases, especially in elderly males [1], with a specific mortality rate of 5% [2]. Theories based on histopathologic and clinical data suggest that it is caused by a combination of cardiovascular disease, hemodynamic compromise, and decreased function of mucosal barrier caused by gastric acid reflux, although the exact pathophysiology is still unclear [1,3]. Management is primarily medical, based on nothing through the mouth status (Nil per os or NPO), proton pump inhibitor (PPI) and treatment of underlying comorbidities. Surgical intervention may be necessary in case of acute complication (esophageal perforation) and delayed complication (esophageal strictures refractory to balloon dilatations) [2,4]. We present a case of acute esophageal necrosis in a 70-year-old female with cardiovascular comorbidities with a new-onset arrhythmia.

## Case presentation

A 70-year-old female patient was admitted to the intensive care unit following two episodes of hematemesis. Her medical history included diabetes mellitus and stented ischemic heart disease. She had no history of malignancy, alcohol abuse, or ingestion of corrosive agents.

On examination, she was conscious but tachycardic, with a heart rate of 140 beats per minute. She was normotensive at 120/60 mmHg, polypneic with a respiratory rate of 25 breaths per minute, and had a pulse oxygen saturation of 95%. Abdominal examination revealed epigastric tenderness, while digital rectal examination was unremarkable. Pulmonary and cardiac auscultation were normal. Electrocardiogram revealed inaugural complete atrial fibrillation.

Biological tests showed an elevated C-reactive protein (CRP) level of 44.7 mg/L, leukocytosis (14×10⁹/L), plateletosis (44x10⁹/L), acute kidney injury was noted, with a creatinine level of 1.93 mg/dL and a glomerular filtration rate of 25 mL/min/1.73m². Blood glucose level was 9.4 mmol/L (1.7 g/L). Hemoglobin level was normal (12.3 g/dL) as was prothrombin time (80%), cardiac troponin (0.03 ng/ml) and liver enzymes. The cyto-bacteriological examination of the urine revealed urinary tract infection with the presence of gram-negative bacteria. The culture confirmed Escherichia coli, antibiogram showed good sensitivity to antibiotics.

**Figure 1:**
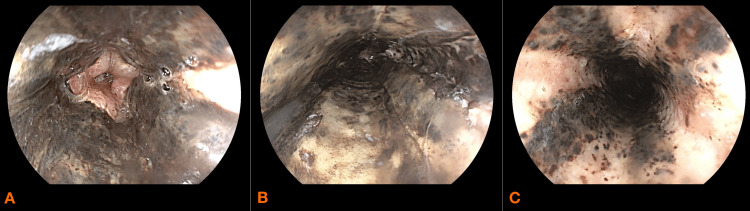
Esophageo-gastro-duodenoscopy performed on day one of admission showing necrotic esophagitis A: lower third of the esophagus B: middle third of the esophagus C: upper third of the esophagus

**Figure 2:**
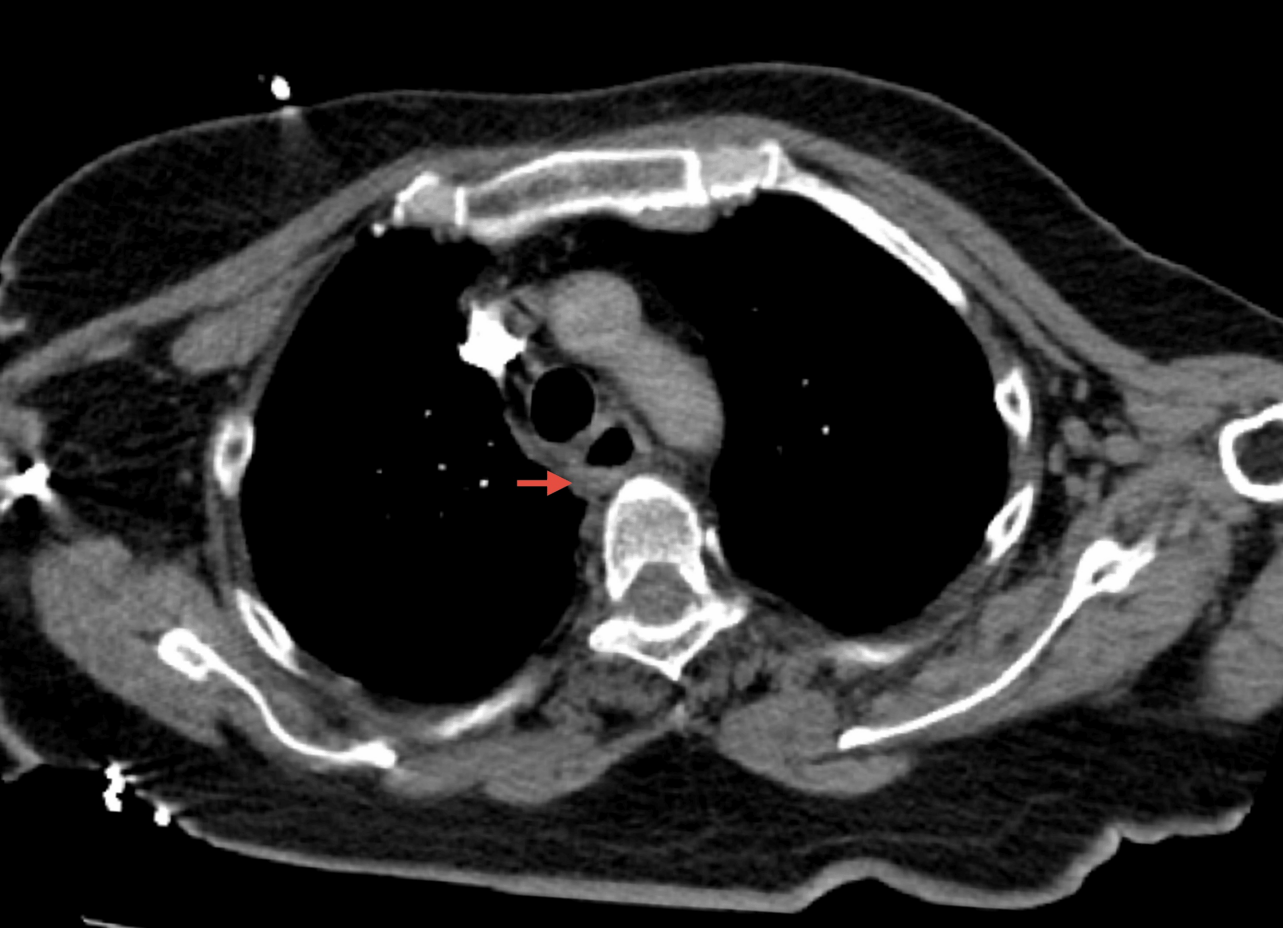
Chest CT scan (axial) revealing thickening of the esophagus (red arrow) without signs of perforation

An esophageo-gastro-duodenoscopy (EGD) was performed on day one which revealed circumferential blackening of the middle and lower third of the esophagus that stopped abruptly at the gastroesophageal junction (Figure 1A, 1B, 1C) with gastritis and Forrest III ulcers with blood in the stomach. A CT scan was performed and showed esophageal thickening and edema with no signs of perforation (Figure 2). 

The treatment included fluid resuscitation, oxygen therapy, continuous high-dose intravenous omeprazole (8 mg/h) following an 80 mg bolus, total parenteral nutrition while maintaining NPO status meaning the patient was not allowed to ingest any food liquids or medications by mouth, and antibiotic therapy with ceftriaxone. Her clinical condition remained stable, with no recurrence of hematemesis. The biological evolution was favorable with a decrease in CRP level and normalization of renal function. 

**Figure 3:**
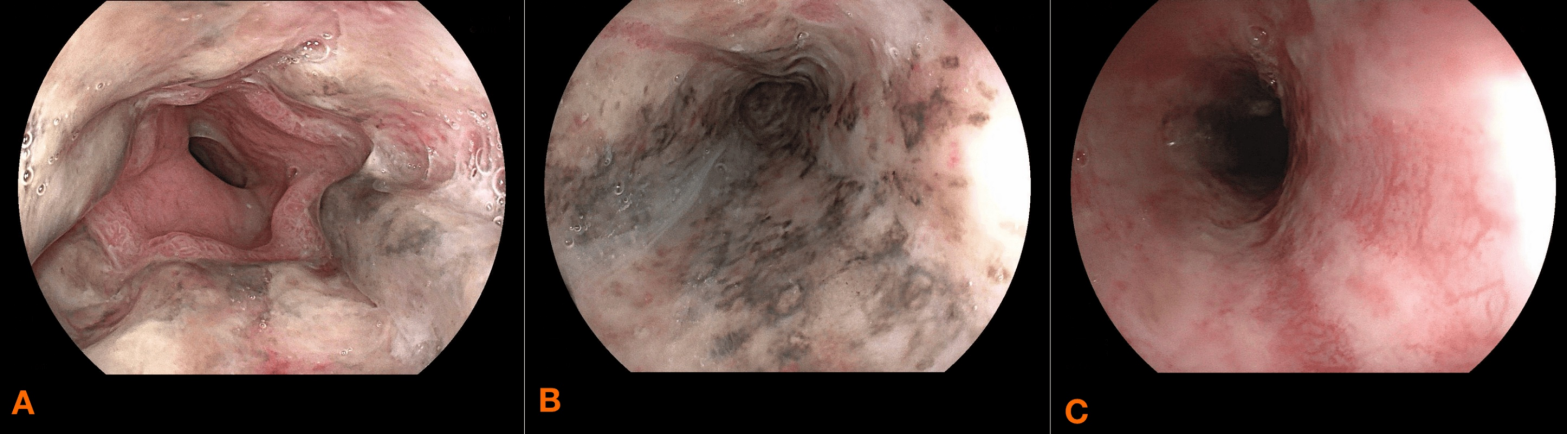
Esophageo-gastro-duodenoscopy performed on day two showing improvment of necrotic lesions A: lower third of the esophagus B: middle third of the esophagus C: upper third of the esophagus

**Figure 4:**
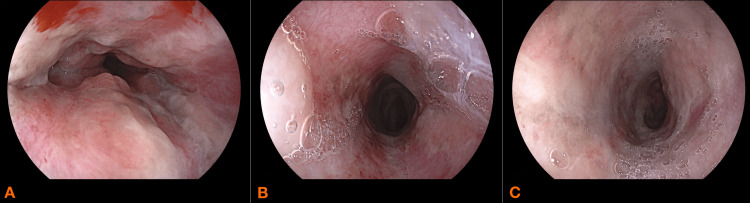
Esophageo-gastro-duodenoscopy performed on day seven showing disparition of necrotic lesions A: lower third of the esophagus B: middle third of the esophagus C: upper third of the esophagus

She underwent repeat EGD at 48 hours and at day seven after admission, both of which demonstrated good progression in the healing of the initial lesions (Figure 3A, 3B, 3C, and Figure 4A, 4B, 4C). Anticoagulation therapy was initiated on the seventh day using subcutaneous enoxaparin, administered at a dose of 40 mg daily. On day 14 fluid oral feeding was reintroduced which she tolerated well.

On day 20 after admission she developed a nosocomial urinary tract infection. Renal function remained normal, she was put on broad-spectrum antibiotic therapy with a combination of piperacillin-tazobactam and amikacin. Despite optimized treatment and resuscitation measures her condition deteriorated further, leading to septic shock requiring vasoactive drug support. Anticoagulation therapy was dismissed due to severe thrombocytopenia. Ultimately, she succumbed to septic shock and passed away on day 24 after admission.

## Discussion

Acute necrotizing esophagitis was first described in 1990 by Goldberg et al. [1] and is a rare and life-threatening clinical entity. The pathogenesis remains unknown, main theories based on histopathologic and clinical data suggest that it is caused by a combination of chronic vascular disease, hemodynamic compromise, and reduced effectiveness of the mucosal barrier and impaired reparative mechanisms resulting from gastric acid reflux, although exact pathophysiology is still unclear [2,3]. The prevalence ranges from 0.1% to 0.3%, with an average onset age of 60 years. It is more commonly observed in males [3,4].

Potential risk factors for developing AEN include diabetes mellitus (noted in approximately 40% of cases), hypertension, cardiovascular diseases (noted in 12%), hypercoagulable state, chronic kidney and liver disease, malnutrition and hypoproteinemia, alcohol abuse (15%), presence of malignancies (10%) and gastroesophageal reflux disease [2,5-7]. In our case, the patient had a history of stented heart disease, and although she was being treated for diabetes, she did not experience hyperglycemia or ketoacidosis during her hospitalization.

A recent case has documented arrhythmia in an elderly patient with acute esophageal necrosis [5]. In our case, inaugural arrhythmia was a significant cardiac event that may have contributed to the development of AEN. This suggests that the early onset of arrhythmia might have led to hemodynamic instability, potentially reducing blood flow to the esophagus and playing a role in the necrotic process.

Clinical symptoms typically include upper gastrointestinal bleeding, manifesting as hematemesis and melena in 90% of cases. Other possible symptoms include epigastric and abdominal pain, vomiting, dysphagia, and nausea, though some cases may remain asymptomatic [2,4].

Endoscopic appearance may vary, it is characterized by partial or complete circumferential black discoloration of the esophageal mucosa, which abruptly terminates at the gastroesophageal junction, as observed in our patient. In most cases, it is confined to the distal esophagus but it may extend to involve entire esophagus [2,4]. Localisation of the lesions may be explained by the poor blood supply and the poor collateral support of the distal third of the esophagus [6].

When the diagnosis is uncertain, esophageal biopsies can be useful, revealing mucosal necrosis, necrotic debris, and the absence of squamous epithelium, with potential involvement of the submucosa and muscularis propria [3,8]. In our case, biopsy samples were not collected due to mucosal fragility and the risk of perforation.

In case of diagnostic uncertainty or suspicion of esophageal perforation, imaging can be performed, revealing air in the mediastinum [7].

Management involves fluid resuscitation to restore hemodynamic stability, supportive blood transfusion in case of significant anemia, antibiotics in case of esophageal infection and intravenous PPI to reduce local injury, while patient should be placed on an NPO rest [6,9,10]. Correcting coexisting clinical conditions and patient’s comorbidities is mandatory to improve patient outcome [10,11]. 

Mucosal healing should be assessed by repeat endoscopic examinations, as the reversible nature of the condition is a positive prognostic factor for complete resolution [6]. 

Acute complications include esophageal perforation (5%) with mediastinal infection and abscess which may occur during the first days after admission [7,10]. Surgery is the primary treatment of perforation and involves mediastinal lavage and abscess decortication, followed by delayed esophageal repair [6]. In some cases, an initial emergency esophagectomy followed by esophageal reconstruction may be performed [12,13]. Direct closure of the esophageal tissue is considered unsafe, and reconstruction is typically delayed [14].

The long-term risk of developing dysphagia due to esophageal strictures or stenosis is 18%, often necessitating endoscopic dilatations [4,6,15]. In cases where balloon dilatation sessions are unsuccessful, a subtotal esophagectomy combined with esophagogastrostomy may be performed [15]. 

The prognosis for AEN is extremely poor, with an overall mortality rate of around 30%, primarily due to underlying medical comorbidities [12,14]. Mortality rate specifically associated with AEN is around 5% [16]. Negative risk factors include esophageal perforation, diabetic ketoacidosis and sepsis [6,15]. In our case, the patient experienced no further episodes of hematemesis and showed good endoscopic improvement. However, mortality was ultimately due to septic shock.

## Conclusions

Black esophagus is a rare, life-threatening condition marked by circumferential black discoloration of the esophageal mucosa. Although the pathogenesis remains unknown, acute ischemia seems to be the most frequent cause. Treatment is urgent and includes hemodynamic stabilization, gastric acid suppression, NPO order and correction of the underlying medical conditions. High-dose intravenous PPI should be initiated to reduce acute and long-term complications and can be changed to oral form after improvment. Esophageal perforation is the most dangerous complication and requires urgent surgery. Esophageal stricture is the most frequent sequelae in survivors. It's essential for physicians to be aware of this entity, as early identification and prompt and aggressive management can enhance survival rates. Inaugural arrhythmia may be a new risk factor for acute necrotizing esophagitis if there will be more cases reported in the future.
